# Cultural Adaptation of an Evidence-Based Dyadic Post Diagnosis Support Intervention for African American Dyads

**DOI:** 10.1093/geroni/igaf122.4092

**Published:** 2025-12-31

**Authors:** Silvia Orsulic-Jeras, Lauren Parker, Kalisha Bonds Johnson, Donna Salaam, Zoe Fete

**Affiliations:** Benjamin Rose, Cleveland, Ohio, United States; Johns Hopkins Bloomberg School of Public Health, Baltimore, Maryland, United States; Emory University, Atlanta, Georgia, United States; Benjamin Rose, Cleveland, Ohio, United States; Benjamin Rose, Cleveland, Ohio, United States

## Abstract

African American older adults are twice as likely to be affected by dementia than non-Hispanic whites. They report unique cultural, structural, and social challenges that can limit access to and engagement with supportive care interventions. SHARE for Dementia is an evidence-based, early-stage dyadic intervention designed to support persons living with dementia (PLWDs) and their care partners in making informed care planning decisions. This presentation describes the first cultural adaptation of SHARE for African American care dyads using a two-phase, community-engagement approach. Phase 1 consisted of a review of the original SHARE protocol by an Advisory Committee (AC; n = 8) comprised of dementia caregiving and subject matter experts. The AC reviewed existing SHARE materials, identified cultural considerations and priorities needed to improve relevance, accessibility, and acceptability. Using a similar methodology, the second phase convened focus groups of African American caregivers and early-stage PWLDs (n = 11). A thematic analysis revealed the following themes: medical mistrust, stigma, lack of dementia education, importance of faith-based support, complex family dynamics, and familial support with future care. Based on these findings, Version 1 of SHARE was developed. Using an iterative approach, both groups reconvened to evaluate V1, yielding refinements that guided development of Version 2, a testable, culturally tailored SHARE. Intervention modifications were systematically documented using the Framework for Reporting Adaptations and Modifications (FRAME) Model. This presentation addresses critical barriers to care planning engagement in African American dyads and lays the groundwork for future testing to determine preliminary efficacy of the culturally adapted SHARE.

